# Measuring football fever through wearable technology

**DOI:** 10.1038/s41598-026-36182-1

**Published:** 2026-02-05

**Authors:** Timo Adam, Jonas Bauer, Christian Deutscher, Christiane Fuchs, Tamara Schamberger, David Winkelmann

**Affiliations:** 1 Faculty of Business Administration and Economics, Bielefeld University, 33615 Bielefeld, Germany; 2 Faculty of Sports Science, Bielefeld University, 33615 Bielefeld, Germany; 3 Institute of Computational Biology, Helmholtz Zentrum München, Neuherberg, Germany

**Keywords:** Fan involvement, Football fever, Smartwatch data, Vital parameters, Wearable technology, Physiology, Psychology, Psychology

## Abstract

Football is the world’s most popular sport, evoking strong physiological and emotional responses among its fans. Yet, the specific reactions to fan involvement have received little attention in the literature. In this paper, we quantify the resulting physiological responses through a unique case study from professional football: the 2025 cup final of the German Football Association (DFB) between first-division club VfB Stuttgart and third-division club Arminia Bielefeld. We collected high-resolution smartwatch data, including heart rate and stress level, from 229 Arminia Bielefeld fans over approximately 12 weeks, complemented by survey responses on identification with the club, match attendance, and personal characteristics from a subset of 37 participants. By combining physiological data with survey information, we analyse variations in emotional arousal across individuals and contexts, measured by physiological reactions to the cup final. This approach provides rare, data-driven insights into the *football fever* that captivates fans during high-stakes competitions. Furthermore, we compare the vital parameters recorded on the day of the match with baseline levels on non-matchdays throughout the entire observation period. Our findings reveal pronounced physiological responses among fans, beginning hours before the match and peaking at kick-off.

## Introduction

For fans, football is an emotional ride — full of joy, frustration, hope, and pride — whether they are cheering in the stands, watching from home, or keeping up with updates on their phones. This collective enthusiasm fuels a thriving football economy, from ticket sales and merchandise to media rights and sponsorship deals^1^. Unsurprisingly, the cultural and commercial significance has inspired a growing body of research that explores football as both an emotional experience and a social phenomenon^2,3^. This paper seeks to quantify emotional arousal through physiological responses in a unique case study in German professional football, examining their intensity, variability, and underlying drivers. It draws on data from fan engagement during the 2025 cup final in Germany, which was arguably the most important match in the history of Arminia Bielefeld, one of the two participating clubs.

The cup of the German Football Association (*DFB-Pokal*) is a prestigious national competition featuring 64 teams. The tournament follows a single-elimination format and includes all clubs from the first two national divisions, along with the winners of regional cup competitions. Traditionally, the cup final is staged at the end of each season at Berlin’s *Olympiastadion*, the largest stadium in Germany’s capital, and constitutes a nationwide spectacle watched by millions of fans. On May 24, 2025, VfB Stuttgart, the runner-up of the previous national league season and strong favourite, faced off against third-division club Arminia Bielefeld. This marked only the fourth occasion since the competition’s inception in 1935 that a third-division club reached the final. For Arminia Bielefeld, their first appearance in the cup final was an extraordinary achievement that captured national attention and enthused the entire region surrounding Bielefeld for weeks.

The literature documents that fans often develop strong psychological bonds with their team, with concepts such as team identification and identity fusion describing how fans feel “at one” with their club^4^. This bond amplifies emotional reactions to match events, triggering substantial physiological responses. Exposure levels in football stadiums can exceed limits of permissible sound with the potential risk of changes in post-match hearing^5^. In addition, research has recorded increases in spectators’ heart rate, blood pressure, and stress hormones during high-stakes matches^6^. Fans who are firmly fused with their team experience the highest release of stress hormones when under game stress^4^. In extreme cases, the cardiovascular strain of spectating can be severe, leading to an increased incidence of cardiac emergencies^7–9^. Furthermore, the role of the auditory and social environment in shaping spectators’ emotional experiences is emphasised. Specifically, factors such as stadium soundscapes and crowd acoustics influence spectators’ observable emotional reactions, showing how the stadium experience differs from viewing matches on television^10^. Notably, the communal rituals surrounding matches can also evoke powerful shared emotions: For example, the excitement of Brazilian football fans (measured via heart rate arousal) has been shown to not peak during the match itself but during pre-match fan rituals, with only goal celebrations matching that pre-match high^11^. Summarising, this literature underscores how profoundly sports fandom engages both mind and body, providing a foundation for quantifying fans’ physical and emotional responses in our case study.

This paper examines the emotional arousal, measured by physiological responses, of sports fans to events involving their favourite club. Unlike the studies discussed above, it utilises smartwatch data to track more than 200 fans over several weeks, not just the day of the match. This enables us to isolate the effects of the match from other dynamics. The 2025 cup final serves as a natural experiment in our study, given its exceptional importance to Arminia Bielefeld fans. In collaboration with the *Wissenswerkstadt Bielefeld*, we recorded vital parameters from Arminia Bielefeld fans over a 12-week period before, during, and after the cup final using *Garmin* smartwatches. Additionally, participants were surveyed to assess their identification with Arminia Bielefeld, attendance at the cup final, and personal characteristics. We received responses to the questionnaire from a subset of participants, allowing us to link survey information with the smartwatch data. This linkage enables us to draw conclusions about the emotional arousal of sports fans based on their characteristics and factors such as the location where they watched the match. Our study is relevant to sports science, psychology, and wearable-technology research, as it provides rare large-scale physiological measurements (of fan responses) over an extended period. It also offers valuable insights for sports management and public health by demonstrating how match context and viewing environments influence physiological strain among fans.

## Data collection

Our study aims to explore the emotional arousal of Arminia Bielefeld fans to the cup final. To this end, fans who intended to watch the match were recruited and invited to share their physiological data with us. After the final, participants were asked to complete a survey and provide additional information to enable a more in-depth analysis. This section outlines the procedure for collecting smartwatch data and describes the survey distributed to participants, along with summarised responses.

### Smartwatch data

Our study includes 229 adult Garmin smartwatch users (aged 18 years and older), all of whom self-identified as Arminia Bielefeld fans, ensuring that an identification with the club can be expected. Participants were recruited through local and national media reports, including the club’s website, and were required to register on Bielefeld University’s website. No further selection was applied among those who registered, and fans were not reimbursed for their participation. To contextualise the findings from the cup final with respect to vital parameters observed on *regular* days, data were collected over an extensive period, commencing on May 14, 2025, 10 days before the cup final, and concluding on July 31, 2025. Here and in the following, *regular* days are days without an official match of Arminia Bielefeld and without local holidays. The prolonged observation period enabled us to analyse the day of the cup final in relation to general physiological and behavioural patterns. Participants consented to provide their data for the duration of the study by synchronising their smartwatch with the corresponding smartphone application. The number of active participants slightly varied over time, as not all fans contributed data throughout the entire observation period; specifically, we received data from 194 participants during the cup final itself. Data protection and data consent declarations were developed in collaboration with the *Competence Centre for Research Data* at Bielefeld University and in accordance with relevant institutional, national, and international guidelines and regulations. Before data collection, informed consent was obtained from all participants, including consent to publish analyses of anonymised data.

**Fig. 1:**
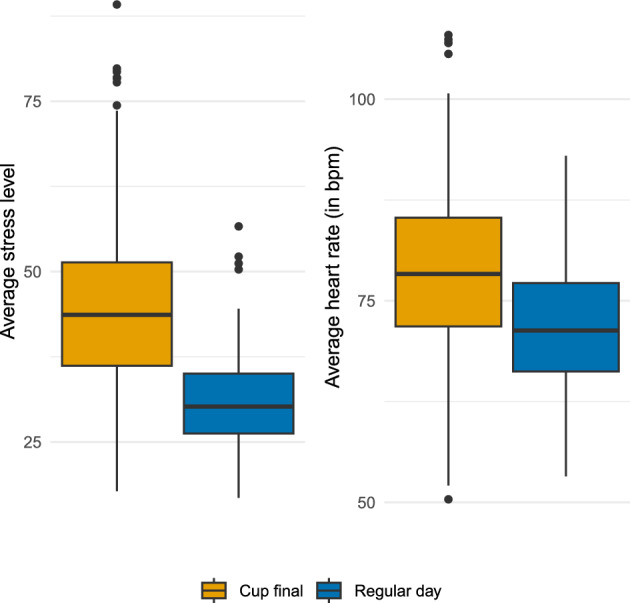
Boxplot of the average stress level (scale from 0 to 100, left) and average heart rate (bpm, right) per individual participant across time points for the day of the cup final (May 24, 2025; orange) and regular days within the observation period (May 14 to July 31, 2025; blue).

The smartwatch records various sports-related metrics, including active seconds, motion intensity, and hourly steps. For this study, we primarily focus on the heart rate and stress level as indicators of fans’ emotional arousal to the match. This choice is consistent with previous research demonstrating the suitability of these variables for capturing emotional responses^12^. The heart rate is the frequency of heart beats per minute (bpm) and is recorded in 15-second intervals. It is measured by an optical sensor on the back of the device. This sensor detects the cycles of pulsing blood flow, i.e. heartbeats. In contrast, stress levels are measured by combining heart rate with heart rate variability, i.e. the variability between consecutive heartbeats, and personal factors to define individual stress and relaxation states. Notably, the stress level is not recorded when the person wearing the smartwatch is highly active (e.g. during exercise), as physical exertion itself induces substantial heart rate variability. Stress levels are expressed on a scale from 0 to 100, with 0 representing no stress and 100 indicating maximum stress and are recorded in three-minute intervals. Descriptive statistics on both stress levels and heart rates are provided in Table 1 and Fig. 1 below.

### Survey of participants

The measured vital parameters of fans are likely influenced by individual characteristics, fan- and match-related factors. To capture these variables, we distributed a survey (full questionnaire provided in the Supplementary Material) to 95 study participants who had consented to further contact after an updated data privacy statement was provided. The survey was structured into three main categories: (1) personal characteristics, (2) match-related variables, and (3) cup final-specific questions.

Given the established link between age and heart rate^13,14^, we incorporated personal characteristics within category (1) of the survey. Beyond age and gender, we expect the degree of identification with Arminia Bielefeld to influence fans’ physiological responses during the match. A dedicated fan who regularly attends matches of Arminia Bielefeld in the stadium may exhibit a different physiological reaction to match dynamics compared with someone who rarely attends in person. We therefore asked for club membership status and the number of matches attended in person during the 2024/25 season as measures of identification with the club.

Under category (2) of the survey, we asked participants where they had followed the cup final. Research in basketball, for instance, has shown that in-person spectatorship produces greater group physiological synchrony and more intense “transformative” experiences than viewing in a small remote group^15^. Those who reported watching the match (either on TV, at a public gathering, or in the stadium) were also asked whether they had consumed alcohol^16^. Category (3) applied exclusively to respondents who attended the final in the stadium. These participants were asked about their arrival time in the host city to capture potential travel-related impact on the vital parameters. Media reports indicated that overcrowding at stadium entrances caused delays, potentially evoking feelings of danger, anxiety, or frustration, which could in turn influence physiological parameters. Accordingly, we asked participants when they joined the entrance queue and how long the entry process lasted. In addition, participants were asked about betting behaviour related to the cup final; however, no one reported having placed a bet.

The survey was distributed to 95 study participants, of whom 37 responded within the requested 16 days: 17 female and 20 male, aged 18 to 63 (average age 38.7 years). Of all participants, 33 were employed, with 30 of them starting work between 6 and 9 a.m. The end of a typical workday for these 30 participants exhibited a much higher variation, occurring between 12:30 and 7 p.m. However, 20 participants indicated that their usual workday ended between 4 and 6 p.m. Among the respondents, 18 (48.6%) were Arminia Bielefeld members and 14 (37.8%) were season ticket holders; 12 participants were both.

Except for one survey participant, all of them watched the cup final: 20 attended the match in the stadium, five joined public gatherings, and 11 watched the match on TV. 50% reported consuming alcohol during the cup final, with a notably higher rate of 65% among those who attended in the stadium. Furthermore, 77.8% of club members and 78.6% of season ticket holders attended the match in the stadium. Additionally, all 11 fans who attended more than 10 matches of Arminia Bielefeld in stadiums during the 2024/25 season also watched the cup final in the stadium in Berlin in person, compared with less than 50% of those who attended only 1–10 matches during the season. The cup final took place on a Saturday evening, starting at 8 p.m. Of the 20 participants who attended the match in person, nine arrived in Berlin on Friday or earlier, while the remaining 11 came on the day of the match. Eighteen fans participated in the fan festival held before the match in a central location in Berlin.

## Results: fans’ emotional arousal to the cup final

Collecting data from smartwatches worn by study participants enables us to investigate their emotional arousal to the 2025 cup final by analysing the physiological responses — specifically stress levels and heart rates — during the match and comparing these with patterns observed on regular days. In this section, we (1) present summary statistics on participants’ stress levels and heart rates, (2) consider patterns of vital parameters on the day of the cup final in comparison to the entire observation period, and (3) take a closer look at the course of the cup final itself. The discussion of our findings is left to the subsequent section.

### Summary statistics

**Table 1:** Summary statistics of stress levels (scale from 0 to 100) and heart rates (bpm) for individual participants, measured at single time points, on the day of the cup final (May 24, 2025) and on regular days within the observation period (May 14 to July 31, 2025).

Variable	Time window	Minimum	1. Quartile	Median	Average	3. Quartile	Maximum
Stress level	Cup final	0.0	21.0	40.0	44.2	67.0	100.0
	Regular day	0.0	16.0	24.0	31.1	43.0	100.0
	Overall	0.0	16.0	25.0	31.3	43.0	100.0
Heart rate (bpm)	Cup final	35.0	64.0	77.0	78.7	91.0	181.0
	Regular day	27.0	58.0	68.0	70.9	80.0	243.0
	Overall	27.0	58.0	69.0	71.0	81.0	243.0

Table 1 presents summary statistics of the individual stress levels and heart rates. One observation represents the corresponding value of a single participant at a specific time point. We divided the data into the day of the cup final and regular days. Stress levels on both the cup final day and on regular days covered the complete scale (0 to 100). However, all remaining descriptive statistics for stress levels were higher on the day of the cup final, with the average stress level (44.2) being increased by approximately 41% compared with regular days (31.3). Similarly, heart rate statistics were generally elevated on the day of the cup final, with the exception of the maximum value. Moreover, we have calculated the 95% confidence interval for the mean stress level across the 78 regular days. This interval (28.1 to 35.6) did not include the average stress level on the day of the cup final, indicating a statistically significant increase. Similarly, we calculated the 95% confidence interval for the average heart rates on regular days. As with stress levels, this interval did not cover the cup final average heart rate (78.7). Therefore, the average heart rate on the day of the cup final was significantly higher than on regular days.

Figure 1 illustrates the distribution of the average stress levels (left panel) and average heart rates (right panel) for each participant across time points using box plots comparing the day of the cup final to regular days. We find that both the average heart rate and average stress level were elevated on the day of the cup final. While the maximum average stress level on regular days was approximately 50, values rose to as high as 90 for single individuals on the day of the cup final. Notably, the variation in stress levels among study participants was much greater on the day of the cup final. For the relation between heart rates and stress levels on the day of the cup final and regular days, see Figure S1 in the Supplementary Material.

### Vital parameters over the course of the matchday in light of regular days

**Fig. 2:**
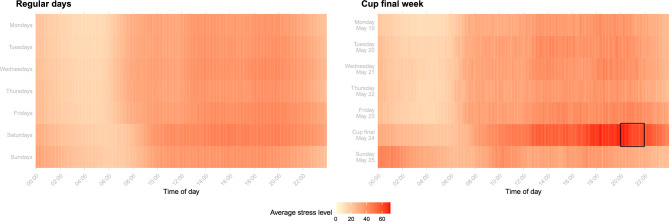
Average stress levels (scale from 0 to 100) across participants and across regular days between May 14 and July 31, 2025 (left panel) and average stress levels (scale from 0 to 100) across participants over the course of the day from May 19 to May 25, 2025 (right panel). The black box highlights the cup final on May 24.

In this section, we compare vital parameters on the cup final matchday with those on regular days within the observation period, thereby providing a basis for understanding how matchdays alter typical physiological dynamics. Figure 2 illustrates the average stress levels across participants and across regular days throughout the study’s observation period (left panel) and average stress levels across participants during the week of the cup final (right panel). Darker colours indicate higher average stress levels. As we observe variation in participants’ stress level patterns depending on the day of the week, we averaged stress levels in the left panel not only across all participants but also across the same weekdays of different weeks. Consequently, the values for regular days represent the average stress levels across all participants over the 12 weeks of data collection and across the respective day of the week. Averages per time point were calculated based on the number of applicable measurements, which varied slightly.

Recurring patterns were evident across weekdays, with stress levels generally lowest at night. Additionally, we observe clear differences between typical weekdays (Monday to Friday) and weekends (Saturday and Sunday). On weekdays, average stress levels started to increase at around 6 a.m., whereas on weekends, they did not rise before 8 a.m. Furthermore, average stress levels on regular Saturday nights were noticeably higher than on weeknights. Overall, Saturdays appeared to be the most stressful days, showing higher average stress levels than other days of the week during time awake, even in weeks without a football match. In contrast, weekdays tended to be less stressful, with average stress levels on Sundays comparable to those observed on weekdays.

We now compare the week of the cup final (right panel) with averages across regular days (left panel). Overall, the two panels show broadly similar patterns for most of the time. However, the day of the cup final, May 24, stands out clearly. On that day, the average stress level was considerably higher than on any regular day, including regular Saturdays, starting from typical wake-up times. Notably, an elevated average stress level was already apparent during the preceding night. It continued to rise over the course of the day, particularly after lunch, and peaked between 6 and 8 p.m., right before kick-off. Although substantial differences in average stress levels were evident during the match, it remained elevated afterwards, exceeding averages observed at any time on regular days.

**Fig. 3:**
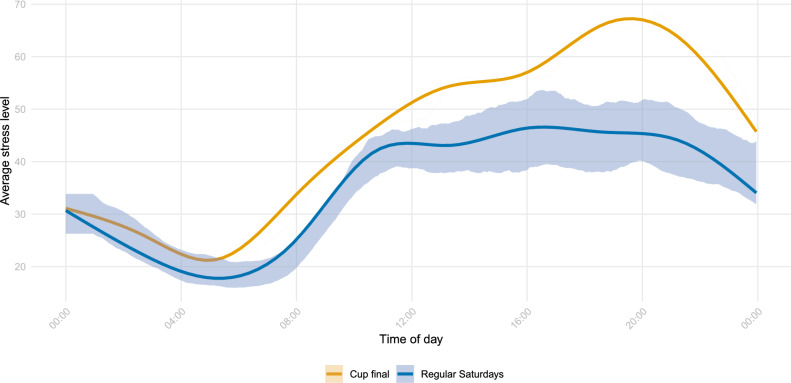
Average stress level (scale from 0 to 100) across participants over the course of the cup final matchday (May 24, 2025; orange) compared with the average stress level (scale from 0 to 100) across participants and across all regular Saturdays within the observation period (May 14 to July 31, 2025; blue). The shaded areas represent the 10th- and 90th-percentiles of averages across participants for regular Saturdays. For the graphical representation, we smoothed averages and percentiles to reduce volatility and increase interpretability.

Next, we evaluate the difference between the Saturday on which the cup final took place and regular Saturdays — the days that were, on average, the most stressful during the week anyway — in more detail. Figure 3 illustrates the average stress levels across participants on the day of the cup final compared with the average stress levels across participants on regular Saturdays, with the latter being averaged across both individuals and multiple Saturdays. In addition, it displays the 10th- and 90th-percentiles across Saturdays: for each Saturday and each time point of the day, stress levels were first averaged across participants, yielding one value per time point and Saturday; the percentiles were then computed across these values at each time point. Similar to our findings in Fig. 2, we observe higher average stress levels on the day of the cup final than on regular Saturdays at every time of the day. This difference was particularly pronounced before kick-off and during the match. Specifically, during the match, i.e. between 8 and 10 p.m., the average stress level was higher by 43% compared with regular Saturdays. Moreover, during the typical time awake, the average stress levels on the day of the cup final exceeded the 90th-percentile of regular Saturdays at every time point.

### Vital parameters over the matchday and the course of the match

The final of the German cup competition on May 24, 2025, attracted considerable national and international attention, primarily due to the participation of third-division club Arminia Bielefeld. Given the exceptional significance of this event for Arminia Bielefeld fans, this section takes a closer look at the cup final itself and the responses of supporters who attended the match. We first outline the course of the cup final, then present fans’ vital parameters over the match period in relation to the uncertainty of the match outcome and across different viewing contexts, and finally consider the impact of the day of arrival in the host city on fans’ stress levels for those who attended in the stadium.

According to pre-match betting odds, provided by a major European bookmaker, VfB Stuttgart, the runner-up of the previous national league season and opponent of Arminia Bielefeld, was deemed the clear favourite. Specifically, translating these betting odds into odds-implied winning probabilities as a predictor for the match outcome^17^, the estimated probability of a VfB Stuttgart victory was 70%, a draw after 90 minutes was about 20%, and an Arminia Bielefeld win was approximately 10%. Although the initial phase of the match was quite balanced, with even promising scoring opportunities for Arminia Bielefeld, VfB Stuttgart scored in the 14th, 22nd, and 27th minutes. This 3-0 lead increased VfB Stuttgart’s odds-implied winning probability to over 95%, rising to more than 97% by halftime. In the second half, VfB Stuttgart scored again in the 67th minute, before Arminia Bielefeld was able to reduce the deficit with goals in the 84th and 87th minutes, eventually resulting in a 4–2 victory for VfB Stuttgart. Despite these being the first goals ever scored by a third-division club in the cup final, they had minimal impact on the odds-implied probabilities: Prior to Arminia Bielefeld’s first goal, the probabilities were 99.5% for a VfB Stuttgart win and 0.25% each for a draw and an Arminia Bielefeld victory. Following the 4–2 scoreline, VfB Stuttgart’s winning probability only slightly decreased to 97.7%, with a 2.0% probability of a draw and 0.3% for an Arminia Bielefeld win.

**Fig. 4:**
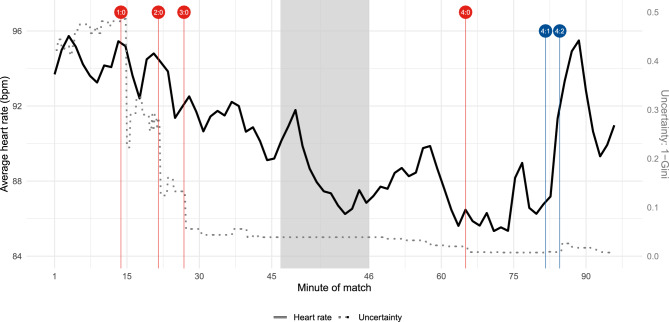
Average heart rate (bpm) across participants over the course of the match (black solid line; left y-axis) and objective uncertainty of the match outcome derived from betting odds measured by $1-\text {Gini}$ (grey dotted line; right y-axis) during the cup final. For the graphical representation, we smoothed the average heart rate to reduce volatility and increase interpretability. The grey shaded area marks the halftime break. Goals are indicated by vertical lines (red: VfB Stuttgart; blue: Arminia Bielefeld), together with the resulting scoreline.

Figure 4 illustrates the average heart rate of those study participants who provided data throughout the cup final, starting from kick-off at 8 p.m. (minute 1) until the end of the added time (around 10 p.m.). To relate fan involvement to uncertainty about the match outcome, the figure additionally depicts the measure $1-\text {Gini}$ to quantify (objective) uncertainty (grey dotted line, right y-axis). The Gini coefficient is calculated based on odds-implied probabilities and ranges from 0 to 1, with higher values of $1-\text {Gini}$ indicating a higher degree of uncertainty. For example, odds-implied probabilities of one-third for each of the three possible outcomes would yield a $1-\text {Gini}$ value of one.

Results presented in Fig. 4 illustrate that the heart rates of Arminia Bielefeld fans were highest during the first 15 minutes of the match, with the average reaching up to approximately 96 bpm. After each goal scored by the opposing team, VfB Stuttgart, we observe a decrease in the heart rate, with the average value falling below 90 bpm until the beginning of the halftime break. The average heart rate increased during the first few minutes of the break; however, when the match resumed, it remained relatively low between 86 and 90 bpm. The average heart rate dropped to its lowest point of the match shortly after the fourth goal by VfB Stuttgart (below 86 bpm around the 70th minute). Especially for the first half, average heart rates aligned with the development of objective uncertainty, represented by $1-\text {Gini}$: Both lines peaked within the first 15 minutes of the match and then steadily decreased until halftime. While the average heart rate declined more gradually, the objective uncertainty based on odds-implied probabilities decreased in steps following each goal. The results in Fig. 4 are particularly interesting for the last 15 minutes of the match, where Arminia Bielefeld scored two goals. While the objective uncertainty of the match outcome remained very low (the value of $1-\text {Gini}$ increased from 0.007 to only 0.026 after the two goals), the average heart rate increased by around 10 bpm and reached similar values after the second goal by Arminia Bielefeld as during the first 15 minutes of the match.

**Fig. 5:**
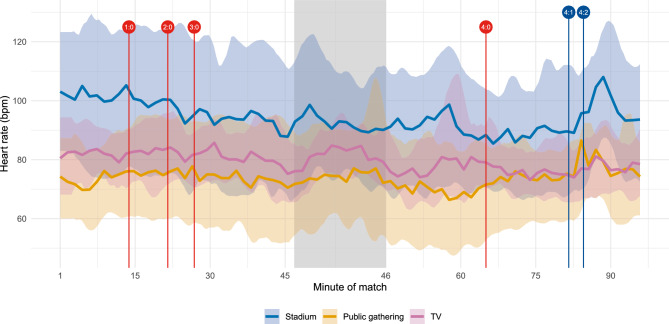
Average heart rate (bpm) across survey participants with the same viewing context over the course of the match (blue: stadium; orange: public gathering; pink: TV). The shaded areas represent the heart rates’ 10th- and 90th-percentiles. For the graphical representation, we smooth averages and percentiles to reduce volatility and increase interpretability. The grey shaded area marks the halftime break. Goals are indicated by vertical lines (red: VfB Stuttgart; blue: Arminia Bielefeld), together with the resulting scoreline.

To further contextualise the fan experience, Fig. 5 shows the average heart rate over the course of the match, while distinguishing between participants who watched the match in the stadium, at a public gathering, or on TV. These values are based on different numbers of participants, determined by the location of the match attendance, and include only those 37 participants who completed the additional survey. The average heart rate was highest for participants in the stadium (average: 94.2 bpm), followed by those watching on TV (79.4 bpm) and at a public gathering (73.8 bpm). On average, heart rates of stadium attendees were 23.1% higher than those of participants watching elsewhere. After the first goal of Arminia Bielefeld, this difference increased to up to 35.8%, with a maximum average heart rate of 108.0 bpm in the stadium. Beyond the location where the match was watched, alcohol consumption was also associated with elevated heart rates: The average heart rate of participants who reported alcohol intake was on average 5.3% higher throughout the match, 7.4% higher during the second half, and even 11.7% higher following Arminia Bielefeld’s first goal.

**Fig. 6:**
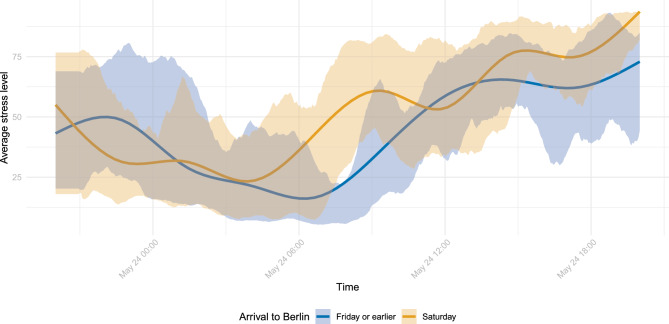
Average stress levels (scale from 0 to 100) across survey participants who attended the final in the stadium with the same arrival in Berlin (orange: Friday (or earlier); blue: Saturday) over the 24 hours prior to kick-off. The shaded areas represent the stress levels’ 10th- and 90th-percentiles. For the graphical representation, we smooth averages and percentiles to reduce volatility and increase interpretability.

For the study participants who attended the cup final in the stadium, we further gathered information on whether they arrived in the host city on Friday (or earlier) or on Saturday, i.e. the matchday. Figure 6 illustrates the average stress levels across these fans, differentiated by the day of arrival, along with the 10th- and 90th-percentiles. For both groups, the average stress level steadily increased over the course of the matchday, peaking just before kick-off. Notably, there were distinct differences depending on the day of arrival. Fans who travelled to Berlin prior to the matchday exhibited higher average stress levels late Friday evening. In contrast, those arriving on Saturday displayed increased average stress levels from 6 a.m. on the matchday, while the increase of the Friday group started around 8 a.m. only. While the average stress levels were relatively similar between 11 a.m. and 2 p.m., fans arriving on Friday or earlier exhibited lower average stress levels from 2 p.m. until kick-off.

## Discussion

The observations presented in the previous section allow us to draw conclusions on the emotional arousal of Arminia Bielefeld fans to the cup final, and compare their physiological responses to those obtained on regular days. For the latter, they show expectable patterns: we find the lowest stress levels when most people are asleep, with increases starting later on weekends than on weekdays, likely due to people sleeping in. Similarly, stress levels on Saturday nights were higher compared to other days of the week, probably because people were more likely to go out. In general, people appeared to be more active on Saturdays than on other days.

Considering the event of the cup final on May 24, our results emphasise the event’s impact on participants’ average stress levels. The day of the cup final appeared to be more stressful for participants even than other Saturdays, most likely due to the emotions surrounding their club’s participation in this outstanding event. Fans who travelled from Bielefeld to Berlin, the host city of the cup final, the day before, and therefore stayed awake for longer than usual, contributed to elevated average stress levels already in the night before the final. In contrast, higher stress levels from typical wake-up times on the matchday might partly be attributed to some fans travelling on this day; it further suggests that fans were already excited about the match several hours before kick-off. Additionally, we observe differences depending on the day of arrival throughout the entire matchday. Potentially, fans staying overnight in Berlin had the opportunity to rest in their hotels. In contrast, those arriving solely on the matchday might have left the host city directly after the match and thus had to remain active the whole day, e.g. by visiting the fan festival of Arminia Bielefeld. In conclusion, fans travelling to the host city of the cup final only on the matchday exhibited higher average stress levels throughout the entire day (see Fig. 6). The peak in average stress levels across all study participants right before kick-off likely reflects both heightened fan involvement and increased fan mobility, such as going to the stadium, public gatherings, friends’ houses, or welcoming guests. However, the observed sustained elevation even after the match was plausibly linked to the intense emotions associated with the match experience.

Comparing physiological responses on the matchday to regular days, we observe a considerably higher variance in stress levels on the matchday, potentially influenced by the different locations where study participants attended the match. These findings suggest that the emotional and physiological arousal associated with watching the match was particularly pronounced in the stadium environment compared with other viewing contexts. This is in line with previous literature suggesting that attending matches in the stadium leads to more extreme physiological responses due to bonding with other fans^18,19^. In addition, interpretation of the heart rate should take into account that alcohol intake can contribute to an acute increase in cardiovascular strain, particularly in emotionally arousing contexts: Elevated heart rates in combination with alcohol are known to increase the risk of arrhythmias and other adverse cardiac events^20^, which may be of clinical relevance during high-stress situations such as sports events.

Focusing on the match itself, the increase in the average heart rate during the halftime break can possibly be associated with increased movement, for example, to get refreshments. Despite the almost hopeless scoreline after the break, it might be that fans maintained hope for a comeback of Arminia Bielefeld, coming with a slight increase in emotional arousal during the first 15 minutes of the second half. However, even before VfB Stuttgart scored their fourth goal in the 67th minute, this involvement among Arminia Bielefeld fans had already diminished. For most phases of the match, our results suggest that fan involvement decreased with a delay following major events in football matches. However, during the final minutes of the match, a considerable difference between subjective uncertainty (indicated by physiological responses) and objective uncertainty (measured by $1-\text {Gini}$) were observed. This suggests that there was more fan involvement than objectively quantified uncertainty about the final match outcome. Still, fans might have overestimated the impact of these two goals on it. A different contributing factor might be pride in the extraordinary event, the first scoring in a cup final by a third-division club.

## Conclusion

This study examines the emotional arousal of football fans to high-stakes matches, using physiological responses as its empirical indicator. Drawing on the natural experiment of the 2025 German cup final, our findings demonstrate that such matches elicit pronounced emotional arousal among fans. Using smartwatch data from more than 200 fans of Arminia Bielefeld, one of the two participating clubs, we observe elevated stress and heart rate levels on the matchday, clearly exceeding baseline values from *regular* weekdays and weekends, i.e. days without an official match of Arminia Bielefeld. These increases were evident even prior to kick-off, peaked during phases of the match perceived as decisive, and remained elevated well into the night after the final whistle, indicating sustained arousal throughout the entire matchday. Physiological responses as a measure of involvement, however, varied by context: stadium attendance, alcohol consumption, and the day of arrival in the host city. Moreover, perceived uncertainty regarding the match outcome and pivotal events within the match, such as goals, further amplified the heart rate of fans. Beyond these findings, the study also demonstrates the potential of wearable technology to capture emotional arousal in large samples and over extended periods, providing continuous and non-invasive measures of physiological reactions in the context of a natural experiment. This physiological approach complements previous research focused on spectators’ external emotional reactions, including the acoustically mediated responses^10^.

Still, our study and its results are subject to limitations. First, we rely on indirect stress measures provided by the smartwatches, which are based on heart rate, heart rate variability, and additional individual factors. Consequently, these measures do not capture all aspects of stress, such as cortisol levels or specific external influences and do not represent a general or clinical definition of stress. Similarly, because we rely on self-identified fandom, we cannot assess emotional attachment as a validated psychological construct, which should be measured in future research using validated attachment scales. Second, the post-study survey included only a small subsample of participants. Because we lack information for the full sample, such as where participants watched the match, we cannot attribute the observed effects solely to the cup final. Findings from the subsample already suggest that external factors, such as the viewing context or fans’ general identification with the club, may also explain elevated heart rates. Other influences, such as crowded environments or responses to others’ anxiety or emotions, may likewise affect fans’ heart rates and stress levels. Therefore, future research should examine additional predictors of the physiological responses reported in our study. Third, although we specifically invited persons who identified themselves as Arminia Bielefeld fans, we did not have concrete measures of fans’ identification with the club for all participants. As the degree of identification with the club is likely to affect the emotional arousal to the game, future research should elaborate on the effect of club identification on the emotional involvement with the games in more detail. Finally, future research could profit from integrating broader physiological variables. Recent advances in wearable technology illustrate the potential of multimodal monitoring. For instance, research has demonstrated that multispectral sensor fusion in smartwatches enables continuous assessment of hydration and sweat loss, offering new insights into physiological reactions during emotionally intense events^21^. Similarly, hybrid wearable patches that combine electromyography with cortisol sensing in sweat can capture both muscular and biochemical stress indicators^22^. Incorporating such multimodal data would enable a richer and more precise characterisation of *football fever*, contributing to a comprehensive understanding of the emotional arousal of fans.

In conclusion, our study shows that exceptional events can trigger *football fever* in fans, manifesting as noticeable physiological responses that are likely driven by their emotional arousal to the team and the game. Due to the potential health implications of such elevated stress levels and heart rates, future studies should investigate these physiological reactions in greater detail and across different types of high-arousal events. Beyond the scope of the present work, our study also opens avenues for examining vital parameters in daily life and within general natural high-pressure contexts.

## Supplementary Information

Supplementary Information.

## Data Availability

Match-related data, including the minutes of goals and resulting scorelines, is publicly available. Data on vital parameters required to reproduce the related figures presented in this study are available from the corresponding author upon reasonable request. Betting odds represent proprietary and confidential company data.
