# Supplementary material for: Measuring football fever through wearable technology
Source: Sci Rep. 2026 Feb 5;16:3866. doi: 10.1038/s41598-026-36182-1 (PMC12877198; doi:10.1038/s41598-026-36182-1)
Supplement: Supplementary file 1 — Supplementary Information. [file 41598_2026_36182_MOESM1_ESM.pdf]

Supplementary material — Measuring football fever through wearable technology

Timo Adam[1,+], Jonas Bauer[1,+], Christian Deutscher[2,*,+], Christiane Fuchs[1,3,+], Tamara Schamberger[1,+], and David Winkelmann[1,2,+]

[1]Bielefeld University, Faculty of Business Administration and Economics, Bielefeld, 33615, Germany
[2]Bielefeld University, Faculty of Sports Science, Bielefeld, 33615, Germany
[3]Institute of Computational Biology, Helmholtz Zentrum München, Neuherberg, Germany
[*]christian.deutscher@uni-bielefeld.de
[+]All authors contributed equally to this work and have been ordered alphabetically.

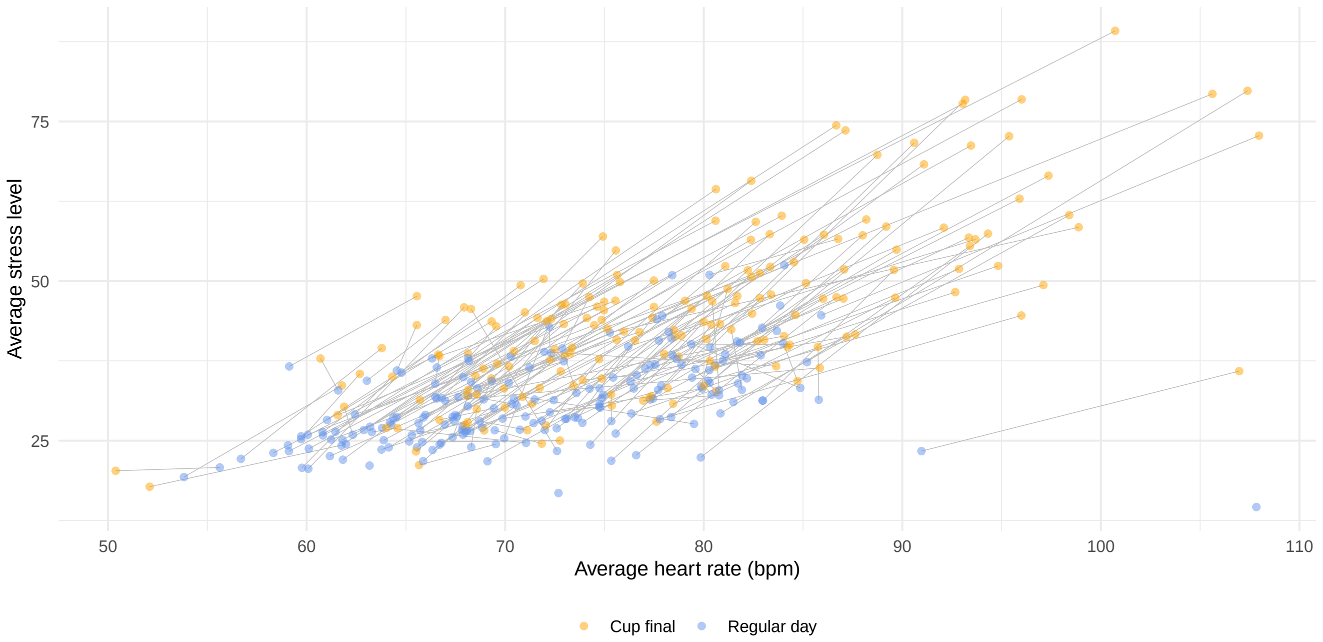

Figure S1. Relation between average heart rate and average stress level for each participant on the day of the cup final (orange) and on regular days (blue).

MUSTER

Bitte so markieren: ☐ ☒ ☐ ☐ ☐ Bitte verwenden Sie einen Kugelschreiber oder nicht zu starken Filzstift. Dieser Fragebogen wird maschinell erfasst.

Korrektur: ☐ ■ ☐ ☒ ☐ Bitte beachten Sie im Interesse einer optimalen Datenerfassung die links gegebenen Hinweise beim Ausfüllen.

Datenverarbeitung

Ich habe die per E-Mail erhaltenen Datenschutzbestimmungen gelesen und stimme der Verarbeitung meiner Daten zu. ☐ Ja

Bitten geben Sie die E-Mail-Adresse an, mit der Sie sich im Mai 2025 zur Teilnahme an der Studie registriert haben. Das ist die Adresse, an die der Link zum Fragebogen versendet wurde. Nur so können wir Ihre Antworten den Daten Ihrer Uhr zuordnen.

MUSTER

MUSTER

Arminia Bielefeld

Welche der folgenden Spiele vom DSC Arminia Bielefeld haben Sie verfolgt?

- ☐ DSC Arminia Bielefeld vs. SV Waldhof Mannheim (3. Liga)
- ☐ DSC Arminia Bielefeld vs. Sportfreunde Lotte (Westfalenpokal)
- ☐ DSC Arminia Bielefeld vs. VfB Stuttgart (DFB-Pokalfinale)

Wo haben Sie das letzte Heimspiel der 3. Liga gegen Mannheim am 17. Mai 2025 überwiegend verfolgt?

- ☐ Im Stadion
- ☐ Mit Videobild (z. B. vor dem Fernseher), in Gesellschaft
- ☐ Mit Videobild (z. B. vor dem Fernseher), alleine
- ☐ Im Radio/Liveticker
- ☐ Sonstiges

Wo haben Sie das Finale um den Westfalenpokal am 29. Mai 2025 überwiegend verfolgt?

- ☐ Im Stadion
- ☐ Mit Videobild (z. B. vor dem Fernseher), in Gesellschaft
- ☐ Mit Videobild (z. B. vor dem Fernseher), alleine
- ☐ Im Radio/Liveticker
- ☐ Sonstiges

MUSTER

Das DFB-Pokalfinale

Wo haben Sie das Pokalfinale am 24. Mai 2025 überwiegend verfolgt?	☐ Im Stadion	☐ Öffentlich (z. B. Public Viewing)	☐ Mit Videobild (z. B. vor dem Fernseher), in Gesellschaft
	☐ Mit Videobild (z. B. vor dem Fernseher), alleine	☐ Im Radio/Liveticker	☐ Sonstiges
Haben Sie Alkohol vor oder während des Spiels konsumiert?	☐ Ja	☐ Nein	☐ Keine Angabe
Wann sind Sie zum Pokalfinale nach Berlin gereist?	☐ Freitag (oder früher)	☐ Samstagvormittag	☐ Samstagnachmittag
Haben Sie das Fanfest vom DSC Arminia Bielefeld am Alexanderplatz besucht?	☐ Ja	☐ Nein	
Hatten Sie Probleme bzgl. der Wartezeit beim Einlass in das Stadion?	☐ Ja	☐ Nein	

Einlassprobleme im Stadion

Zu welcher Uhrzeit haben Sie sich am Einlass in etwa angestellt (hh:mm)?

Wie lange hat der Einlass bei Ihnen etwa gedauert (in Minuten)?

Hier können Sie optional weitere Angaben zur Einlasssituation machen.

Sportwetten

Haben Sie eine monetäre Wette auf den Ausgang des DFB-Pokalfinals getätigt?
- ☐ Ja
- ☐ Nein
- ☐ Keine Angabe

Auf welches Ereignis haben Sie gewettet?
- ☐ Sieg DSC Arminia Bielefeld
- ☐ Unentschieden
- ☐ Sieg VfB Stuttgart
- ☐ Anderes Ereignis (z. B. Anzahl Tore)

Wann haben Sie eine Wette abgeschlossen?
- ☐ Vor Spielbeginn
- ☐ Zwischen Spielbeginn und dem 1:0 für Stuttgart
- ☐ Zwischen dem 1:0 für Stuttgart und der Halbzeit
- ☐ Während der Halbzeit
- ☐ Zwischen Beginn der zweiten Halbzeit und dem 1:4 von Bielefeld
- ☐ Nach dem 1:4 von Bielefeld

In welchem Rahmen haben Sie gewettet?
- ☐ Privat (z. B. mit Freunden)
- ☐ Bei einem Wettanbieter (z. B. Online oder Wettbüro)
- ☐ Woanders

Wie hoch war Ihr Einsatz (in Euro)?

[ ]

Personenbezogene Fragen

Die Beantwortung der folgenden personenbezogenen Fragen ist freiwillig. Sollten Sie eine Frage nicht beantworten wollen, so lassen Sie das entsprechende Feld bitte frei.

Alter

☐ ____________

Geschlecht	☐ Männlich	☐ Weiblich	☐ Divers
Sind Sie aktuell berufstätig?	☐ Ja	☐ Nein	☐ Keine Angabe

Zu welcher Uhrzeit beginnt Ihr Arbeitstag üblicherweise (hh:mm)?

____

Zu welcher Uhrzeit endet Ihr Arbeitstag üblicherweise (hh:mm)?

____

Sind Sie **Mitglied** beim DSC Arminia Bielefeld?	☐ Ja	☐ Nein	
Besitzen Sie eine **Dauerkarte** vom DSC Arminia Bielefeld?	☐ Ja	☐ Nein	
Wie viele Spiele vom DSC Arminia Bielefeld haben Sie in der Saison 2024/25 **im Stadion** besucht?	☐ Keins<br>☐ Mehr als 20	☐ 1-10	☐ 11-20

MUSTER

Einverständnis zur weiteren Kontaktaufnahme

Mit Ihrer Datenspende haben Sie uns geholfen, eine Pilotstudie zum Fußballfieber der Fans vom DSC Arminia Bielefeld während des DFB-Pokalfinales durchzuführen. Auch in zukünftigen Studien möchten wir Zusammenhänge zwischen körperlicher Aktivität, Wohlbefinden und weiteren Merkmalen oder Ereignissen untersuchen. Dürfen wir Sie daher auch zukünftig per E-Mail bzgl. der Teilnahme an einer Studie kontaktieren?

Ich bin einverstanden, dass ich für Folgeumfragen per Mail erneut kontaktiert werden darf. ☐ Ja ☐ Nein

MUSTER
